# *Ginkgo Biloba* Extract Alleviates Methotrexate-Induced Renal Injury: New Impact on PI3K/Akt/mTOR Signaling and MALAT1 Expression

**DOI:** 10.3390/biom9110691

**Published:** 2019-11-03

**Authors:** Iman O. Sherif, Nora H. Al-Shaalan, Dina Sabry

**Affiliations:** 1 Emergency Hospital, Faculty of Medicine, Mansoura University, Mansoura 35516, Egypt; 2 Chemistry Department, College of Science, Princess Nourah bint Abdulrahman University, Riyadh 11671, Saudi Arabia; 3 Medical Biochemistry and Molecular Biology Department, Faculty of Medicine, Cairo University, Cairo 11562, Egypt

**Keywords:** methotrexate, nephrotoxicity, Ginkgo biloba extract, PI3K/Akt/mTOR

## Abstract

Renal injury induced by the chemotherapeutic agent methotrexate (MTX) is a serious adverse effect that has limited its use in the treatment of various clinical conditions. The antioxidant activity of *Ginkgo biloba* extract (GB) was reported to mitigate renal injury induced by MTX. Our research was conducted to examine the nephroprotective role of GB versus MTX-induced renal injury for the first time through its impact on the regulation of phosphatidylinositol 3-kinase/protein kinase B/ mammalian target of rapamycin (PI3K/Akt/mTOR) signaling together with the renal level of TGF-β mRNA and long non-coding RNA-metastasis-associated lung adenocarcinoma transcript-1 (MALAT1) expression. A group of adult rats was intraperitoneally (ip) injected with MTX 20 mg/kg as a single dose to induce kidney injury (MTX group). The other group of rats was orally administered with GB 60 mg/kg every day for 10 days (GB+ MTX group). The MTX increased the serum creatinine and urea levels, renal TGF-β mRNA and MALAT1 expression, in addition to dysregulation of the PI3K/Akt/mTOR signaling when compared with normal control rats that received saline only (NC group). Moreover, renal damage was reported histopathologically in the MTX group. The GB ameliorated the renal injury induced by MTX and reversed the changes of these biochemical analyses. The involvement of PI3K/Akt/mTOR signaling and downregulation of TGF-β mRNA and MALAT1 renal expressions were firstly reported in the nephroprotective molecular mechanism of GB versus MTX-induced renal injury.

## 1. Introduction

Chemotherapeutic drugs are broadly used against various kinds of cancer and their clinical use is associated with the occurrence of unfavorable side effects including many organ toxicities [1]. One of these drugs is methotrexate (MTX) which is a folate antagonist used to treat oncologic and non-oncologic conditions like psoriasis and rheumatoid [2]. The majority of MTX is excreted through the kidney, thus, nephrotoxicity is considered a strong reason which limited its use [3].

Renal impairment developed by the use of MTX was characterized laboratorially by the abrupt rise of serum creatinine and urea with marked elevation of plasma MTX concentration and characterized pathologically by structural changes as degeneration and dilation of renal tubules and glomeruli degeneration [1]. Moreover, the pathogenesis of MTX-induced toxicity involved oxidative stress which played a crucial task in triggering inflammation and apoptosis processes [2,4,5,6].

On the other hand, one of the cellular cascades that is modulated by oxidative stress and inflammation is the phosphatidylinositol 3-kinase (PI3K)/ protein kinase B (Akt) signaling pathway. This pathway is involved in the processes of cell survival, proliferation, and apoptosis [7,8]. One of the proteins regulated by PI3K/Akt is a serine/threonine kinase known as the mammalian target of rapamycin (mTOR). The activation of the phosphorylated mTOR was reported in renal dysfunction and its inhibition resulted in renal protection [9,10,11].

Long non-coding RNAs (lncRNAs) are known as non-protein coding transcripts with the extent of >200 nucleotides [12]. They are the same as protein-coding RNAs with a 5’ cap and poly-A tail. They can participate as RNA in the gene expression regulation and are considered as a remarkable regulator in the pathogenesis of many disorders and biological processes involving differentiation, proliferation, and apoptosis [12,13].

Thus, lncRNAs are now being released as novel biomarkers in many diseases [12,13]. One of the lncRNAs that is well examined is long non-coding RNA-metastasis-associated lung adenocarcinoma transcript-1 (MALAT1) which by its name initially discovered in non-small cell lung cancer and it was found to be expressed in other tissues [14]. Its role in renal injury induced by MTX still has not been identified.

The use of natural products with antioxidant activity gained great attention as safe alternative therapies in attenuation of MTX-induced nephrotoxicity [2,5,6,15]. *Ginkgo biloba* is a popular herbal product used to enhance memory and cognitive function and treated the vaso-occlusive disorders [4]. *Ginkgo biloba* standardized extract (GB) from dried *Ginkgo* leaves contains 24% flavone glycosides and 6% terpenoids and these constituents showed beneficial pharmacological effects in various experimental disease models like antioxidant, anti-inflammatory, and antiapoptotic activities [16,17,18,19].

Furthermore, the renoprotection of GB has been reported versus MTX-induced renal injury through its regulation of oxidant and antioxidant balance [4] thus, this study was performed to illustrate a new protective mechanism of GB via the PI3K/Akt/mTOR/TGF-β signaling pathway in addition to its impact on the expression of MALAT1 in a model of MTX-induced nephrotoxicity.

## 2. Materials and Methods

### 2.1. Animals

The experiment was conducted by following the Guide for Care and Use of Laboratory Animals and was approved by the Ethics Committee of Faculty of Medicine, Mansoura University (NIH publication No. 85-23, revised 2011). Adult male Sprague Dawley rats (250 ± 50 g) were purchased from the Faculty of Veterinary Medicine, Mansoura University and permitted free access to water and standard chow. Controlled conditions were maintained through the whole experiment including temperature 25 °C, and 12 h light/dark cycle.

### 2.2. Experimental Design

Thirty-two rats were divided into four groups and each group included 8 rats as illustrated in Table 1. Methotrexate (MTX, Methotrexate 50 mg, TP, Shanxi PUDE Pharmaceutical Co. Ltd., Shanxi, China) was given as described by previous studies as a single dose of 20 mg/kg intraperitoneally (ip) to induce nephrotoxicity [6,20,21]. While, *Ginkgo biloba* leaf extract (GB, *Ginkgo biloba* 60, GNC, Pittsburgh, PA, USA, Code 194712) was administered by oral gavage 60 mg/kg/day according to the therapeutic dose documented before in various animal models [22,23,24].

### 2.3. Blood and Tissue Sampling

At the end of the experiment, rats were sacrificed under anesthesia. Collection of blood samples were done and serum was separated for renal function tests analysis. Kidneys were removed, one was placed in liquid nitrogen then kept at −80 °C for further biochemical examination and the other kidney was immersed in 10% formalin for histopathology.

### 2.4. Renal Function Tests Measurement

Serum creatinine (Cr) as well as blood urea nitrogen (BUN) were measured using available kits obtained from the Biodiagnostic Company, Cairo, Egypt.

### 2.5. Quantitative Real-Time Polymerase Chain Reactions (q-PCR)

Kidney tissues were homogenized for total RNA isolation using Zymo research (Direct-zol RNA kit). The quality and purity of RNA were assessed and the RNA was kept in −80 °C until used. The cDNA synthesis was performed by using QuantiTect Reverse Transcription Kit from Qiagen using thermal cycler Applied Biosystems StepOne plus (Foster City, USA) for 1 cycle only. For RNA loading control, the GAPDH primers were utilized. Then, the SYBR-green based quantitative real-time polymerase chain reaction (PCR) was done by SensiFAST ™SYBR Hi-ROX Kit. The designed primers used were: TGF-β forward, 5′-TGCGCCTGCAGAGATTCAAG-3′; reverse, 5′-GGTAACGCCAGGAATTGTTGCTA-3′, MALAT1 forward, 5′-ACAGGACTCCATGGCAAACG-3′; reverse, 5′-AACGGATTTGGTCGTATTGGG-3′ and GAPDH forward, 5′-AATGGTGAAGGTCGGTGTGAAC-3′; reverse, 5′-AGGTCAATGAAGGGGTCGTTG-3′. Finally, data analysis was done by using the software version 2.0.1 of Applied Biosystems. The relative quantification of TGF-β and MALAT1 gene expressions were performed using a comparative ΔΔCt method as normalized to GAPDH gene and relative to a control.

### 2.6. Immunoblot Analysis of Phosphatidylinositol 3-Kinase/Protein Kinase B/ Mammalian Target of Rapamycin (PI3K/Akt/mTOR) Signaling Pathway

The ReadyPrepTM protein extraction kit (total protein) from Bio-Rad Inc was employed according to manufacturer instructions and was added to each sample of the homogenized renal tissues. For the quantitative protein analysis, a Bradford Protein Assay Kit was used. Then, loading a 20 μg protein concentration of each sample with an equal volume of 2x Laemmli sample buffer for sodium dodecyl sulfate polyacrylamide gel electrophoresis (SDS-PAGE) were performed. Separated proteins by SDS-PAGE were then transferred to a membrane called an Immobilon membrane from Millipore. The primary antibodies used were: goat polyclonal antibody p-PI3K p85 alpha (Tyr 508), mouse monoclonal antibody p-AKT1 (Ser 473) and mouse monoclonal antibody p-mTOR (Ser 2448) which were quantitated relative to their total antigens t-PI3K, t-AKT, and t-mTOR, respectively (Santa Cruz Biotechnology, INC. Europe). After blocking the membranes for one-hour, primary antibodies were added to membranes and incubated at 4 °C overnight. At room temperature, appropriate secondary antibodies were incubated for two hours. After washing the membranes twice in 1 x TBS-T, the immunoblots densitometric analysis was done to quantify the quantities of the studied proteins versus the control sample by total protein normalization by using Image analysis software (ChemiDoc MP imaging system, Bio-Rad).

### 2.7. Kidney Histopathology Evaluation

Renal tissues were dehydrated in graded ethanol and fixed in paraffin wax. Then sections of 4 μm-thick were examined by a light microscope after staining with hematoxylin and eosin (H&E).

### 2.8. Tissue Injury Score and Glomerular Diameter Estimation

The analysis of the histopathological tissue injury scoring was evaluated as reported by Erboga et al., 2015 [25]. The evaluation was given as the total of the individual score. This score grades from 0 to 3 as follows: 0 indicates no findings, 1 indicates mild, 2 indicates moderate, and 3 indicates severe. The score was given for each of these criteria examined from renal sections as follows: glomerular congestion and degeneration, tubular degeneration, tubular dilatation, cellular vacuolization, and tubular cell swelling. Moreover, the estimation of the glomerular diameter was performed in thirty glomeruli/renal section from each rat as described before by Wen et al., 2013 [26].

### 2.9. Statistical Analysis

The computer software SPSS version 20 was used for data analysis. Values were expressed as Mean ± SD. The differences between groups were assessed by analysis of variance (ANOVA) followed by Bonferroni multiple tests for biochemical analyses and followed by Tukey’s post-test for glomerular diameter. However, the differences among groups in histopathological scoring were evaluated by the Kruskal–Wallis test followed by Dunn’s test to compare all means. When the value of p was less than 0.05, a statistical significance was achieved.

## 3. Results

### 3.1. Effect on Renal Function Tests

**Figure 1:**
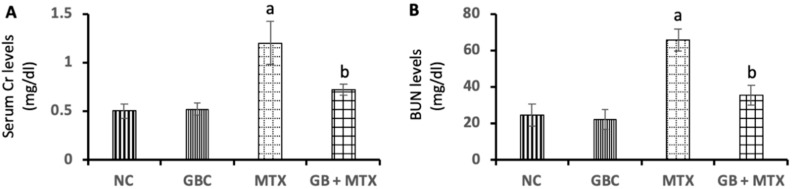
Effect of 20 mg/kg methotrexate (MTX, ip) only and when combined with 60 mg/kg *Ginkgo biloba* extract (GB, orally for 10 days) on the serum creatinine (Cr)(**A**), and blood urea nitrogen (BUN) (**B**) levels in all studied groups. ^a^ *p* < 0.05 versus NC group. ^b^ *p* < 0.05 versus MTX group.

As illustrated in Figure 1, our data showed a marked increase in serum Cr (A) by 140%, and BUN (B) by 168.8% in MT X group in comparison to the NC group. GB treatment ameliorated renal function tests via a marked reduction in serum Cr (40%) and BUN (46%) in comparison to the MTX-treated group (*p* < 0.05).

### 3.2. Effect on Renal TGF-β mRNA Expression

**Figure 2:**
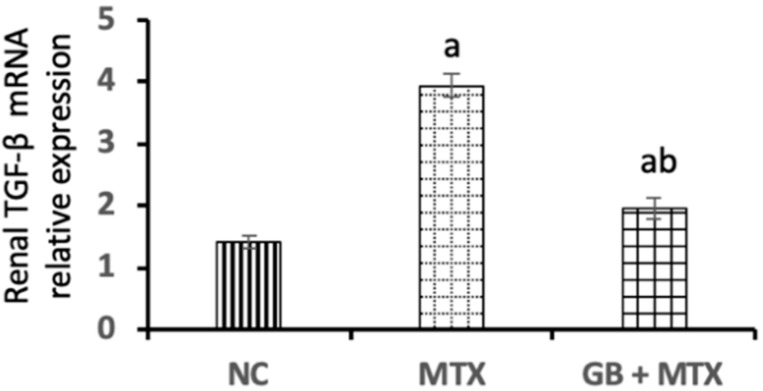
Effect of 20 mg/kg methotrexate (MTX, ip) only and when combined with 60 mg/kg *Ginkgo biloba* extract (GB, orally for 10 days) on transforming growth factor-beta (TGF-β) mRNA expression in renal tissue in all studied groups. ^a^ *p* < 0.05 versus NC group. ^b^ *p* < 0.05 versus MTX group.

In Figure 2, a marked upregulation of TGF-β mRNA level in renal tissue by 1.8-fold was reported in the MTX group compared to the NC group. Administration of GB resulted in a notable downregulation of renal TGF-β mRNA expression by 50.3% when compared with the MTX group (*p* < 0.05).

### 3.3. Effect on PI3K/Akt/mTOR Pathway

**Figure 3:**
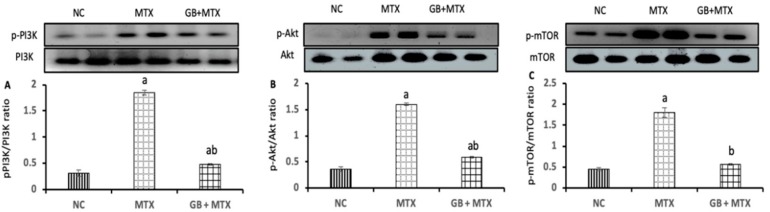
Effect of 20 mg/kg methotrexate (MTX, ip) only and when combined with 60 mg/kg *Ginkgo biloba* extract (GB, orally for 10 days) on the activity of phosphatidylinositol 3-kinase/protein kinase B/ mammalian target of rapamycin (PI3k/Akt/mTOR) signaling pathway. The western blots bands of proteins and its relative expression p-PI3K/PI3k protein (**A**); p-Akt/Akt protein (**B**); and p-mTOR/mTOR protein (**C**) in renal tissue in all studied groups. ^a^*p* < 0.05 versus NC group. ^b^*p* < 0.05 versus MTX group.

To assess the role of PI3K/Akt/mTOR signaling pathway in MTX-induced renal injury and the effect of GB on this pathway, we checked the phosphorylation levels of PI3K, Akt, mTOR in the renal tissue of all experimental groups as presented in Figure 3. MTX induced a significant activation in the phosphorylation of PI3K (**A**) by 4.9-fold with the activation of the p-Akt (**B**), and p-mTOR (**C**) proteins by 3.4-fold and 3-fold, respectively, when compared to the NC group. While, GB treatment inhibited the activation of the p-PI3K by 74%, p-Akt by 63.1%, and p-mTOR by 68.3% in the kidney compared to the MTX group (*p* < 0.05).

### 3.4. Effect on Renal Long Non-Coding RNA-Metastasis-Associated Lung Adenocarcinoma Transcript-1 (MALAT1) Expression

**Figure 4:**
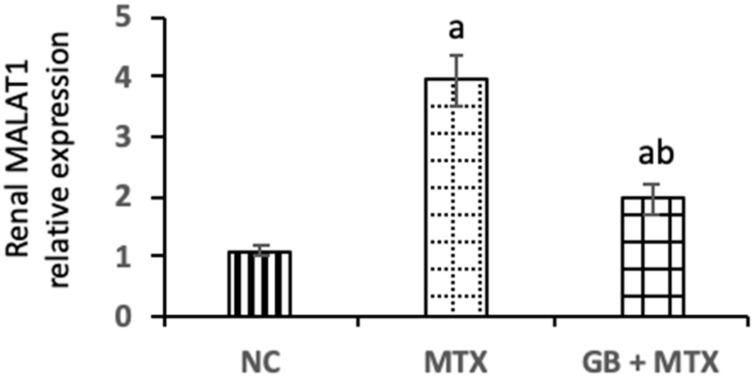
Effect of 20 mg/kg methotrexate (MTX, ip) only and when combined with 60 mg/kg *Ginkgo biloba* extract (GB, orally for 10 days) on long non-coding RNA-metastasis-associated lung adenocarcinoma transcript-1 (MALAT1) expressions in renal tissue in all studied groups. ^a^*p* < 0.05 versus NC group. ^b^*p* < 0.05 versus MTX group.

A marked upregulation of MALAT1 expression by 2.5-fold was observed in the kidneys of MTX injected rats when compared with NC rats (Figure 4). Administration of GB exhibited a notable downregulation of renal MALAT1 expression by 50.1% compared to the MTX group (*p* < 0.05).

### 3.5. Effect on Renal Histopathology

**Figure 5:**
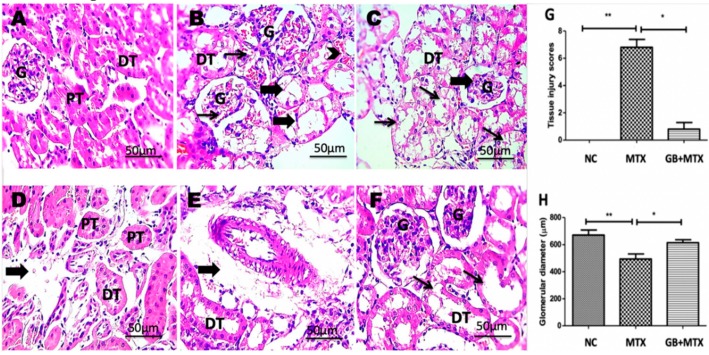
Kidney showing normal histological structure of renal glomeruli (G), proximal (PT) and distal (DT) tubules in NC group (**A**), congested glomeruli (thin arrows) with tubular degeneration and dilation (thick arrows), congested interstitial blood vessels (arrowhead) (**B**), shrunken glomerular tuft (thick arrow) with marked hydropic degeneration in tubular epithelium (thin arrows) (**C**), interstitial edema (thick arrow) (**D**) and perivascular edema (thick arrow) (**E**) in MTX group, tubular dilation (**F**) in GB + MTX-treated group. (**H**,**E**) X: 400. Statistical analysis of tissue injury scores (**G**) and glomerular diameter (**H**) in all studied groups. ** *p* < 0.05 versus NC group. * *p* < 0.05 versus MTX group.

The renal histopathology of all groups was examined and presented in Figure 5. A normal histological structure was detected in the NC group (A). Congested glomeruli with tubular degeneration and dilation, congested interstitial blood vessels, and shrunken glomerular tuft were observed in the MTX group (B–E). The improved renal architecture was seen with GB administration in the GB + MTX group (F).

### 3.6. Effect on Tissue Injury Score and Glomerular Diameter

A significant increase in renal injury score was shown in the MTX group when compared with the NC group and a marked decrease in the injury score was found after GB administration in the GB + MTX group when compared with the MTX group (*p* < 0.05) (Figure 5G). Moreover, MTX induced a marked reduction in the glomerular diameter by 26.5% compared to NC group while a marked rise in the glomerular diameter by 24.6% was observed in the GB + MTX group in comparison to the MTX group (*p* < 0.05) (Figure 5H).

## 4. Discussion

Nephrotoxicity can occur with the administration of various cytotoxic drugs like MTX [2] as the kidney is the organ that is responsible for the excretion of the toxic metabolites [27]. The renal injury induced by MTX may be attributed to its precipitation in the kidney tubules with its metabolite [3,28]. A significant rise in serum Cr and BUN was reported in our study after MTX treatment when compared with NC indicating impairment of renal function and this is a coup with regard to previous studies [2,15,20]. The nephrotoxicity induced by MTX was evident in our histopathological findings including congested glomeruli with tubular degeneration and dilation, congested interstitial blood vessels, shrunken glomerular tuft, increased capsular spaces, and interstitial and perivascular edema and these pathological changes were documented previously [5,25,29].

The GB improved the renal function in the MTX group confirming its nephroprotective effect and this was in harmony with other studies that reported amelioration of the renal function with the GB administration in models of diabetic nephropathy [10] as well as models of induced nephrotoxicity by cisplatin [30], adriamycin [31], and MTX [4].

In our study, a marked upregulation of renal TGF-β mRNA expression after MTX administration in comparison to the NC group was observed. The TGF-β is a cytokine involved in conditions of inflammation, in addition to endothelial dysfunction and used as an indicator of renal damage [32]. Upregulation of TGF-β levels in renal tissues was reported in models of drug induced-nephrotoxicity including doxorubicin [33] and MTX [34].

Moreover, a marked downregulation of renal TGF-β mRNA expression was noticed after GB administration when compared with the MTX group. In the same way other studies documented the suppression of renal TGF-β mRNA expression after using GB in experimental diabetic nephropathy [35,36].

Furthermore, our results revealed a significant upregulation of the PI3K/Akt/mTOR pathway in MTX-treated rats and this may be explained by what was reported about the capability TGF-β in activating the PI3K/Akt/mTOR pathway [37,38]. Several reports demonstrated the involvement of the PI3K/Akt signaling in the pathogenesis of chemotherapy induced-nephrotoxicity like doxorubicin [11], MTX [5], and cisplatin [39,40]. Controversial results were reported in the PI3K/Akt pathway in different experimental models of induced-kidney injury. Some researchers reported its activation in the injured kidney [37,38,39,41,42,43,44] while others reported its downregulation [5,45,46,47,48]. This may be explained by using different animal models, experimental conditions, duration, therapies, phosphorylation sites analyzed, and detection methods in addition to the early and late stages of renal diseases [11,49,50].

Our study is the first to report the involvement of mTOR activation in MTX-induced nephrotoxicity. Also, it was documented that the mTOR is positively regulated by the PI3K/Akt pathway activation [11]. Its activation played a crucial role in the pathogenesis of different models of kidney diseases as diabetic nephropathy (DN), acute renal injury, and obstructed kidney [9,11,38,42]. The results of this study showed that MTX could activate TGF-β/PI3K/Akt/mTOR signaling and this pathway was mechanistically activated in other models of DN [9] and CCl4 induced liver injury [51].

The GB inhibited significantly the PI3K/Akt/mTOR signaling pathway activation in renal tissue which was induced by MTX treatment. A similar effect was seen with GB but in a model of renal fibrosis in DN [10]. Thus, this work reported for the first time the new renoprotective mechanism of GB in MTX-induced nephrotoxicity via regulation of the PI3K/Akt/mTOR signaling pathway.

Recent research has proved an important role of MALAT1 in the pathophysiological conditions, tumor progression, inflammation, liver fibrosis, and diabetic complications [13,14,52]. Interestingly, we found for the first time a marked upregulation of renal MALAT1 expression following MTX injection. Coinciding with our findings, several studies reported higher expression of MALAT1 in the kidney damage induced via different models including streptozotocin-induced diabetes [53], hypoxic induction [54], renal ischemia/reperfusion [55], and lipopolysaccharide-induced renal injury [56,57]. Thus, we could conclude that MALAT1 expression may be involved in the pathogenesis of MTX induced-renal injury.

Recently it was found that MALAT1 downregulation participated in organ protection against various diseases, for example, its downregulation contributed to the improvement in kidney function after duodenal-jejunal bypass [58] and alleviation of myocardial injury induced by saturated fatty acid [59]. Therefore, targeting MALAT1 may be useful as a therapeutic goal in the treatment of diseases [13]. In accordance with this, this study showed downregulation of MALAT1 after GB administration confirming its nephroprotective mechanism versus MTX induced-nephrotoxicity through its impact on MALAT1 expression.

Although in our study we used the GB which contains a mixture of several natural ingredients without showing the ingredient responsible for the most pharmacological effect, this current study presented a new mechanism of action for GB renoprotection that has not been published yet.

## 5. Conclusions

**Figure 6:**
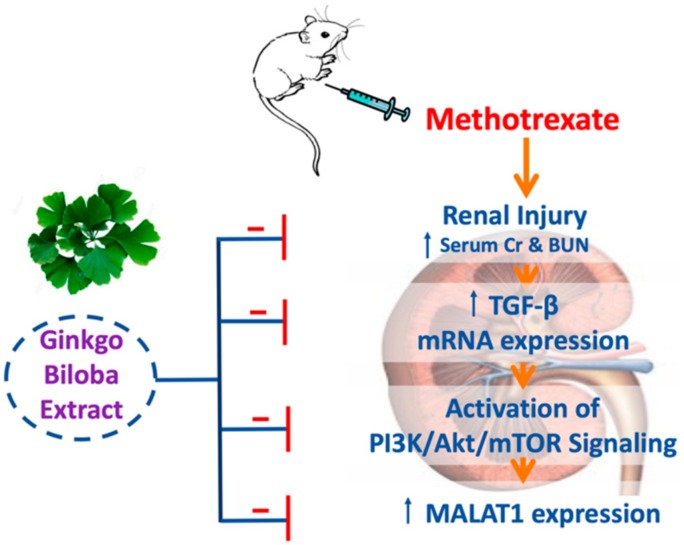
A diagram illustrating the proposed nephroprotective mechanism of GB in a model of nephrotoxicity induced by MTX.

The GB exerted a renoprotective effect against renal injury induced by MTX through multiple mechanisms (Figure 6) including (1) downregulation of renal TGF-β mRNA expression, (2) regulation of the PI3K/Akt/mTOR signaling pathway and (3) downregulation of renal MALAT1 expression. The GB could be used as an adjuvant therapy with MTX to minimize its nephrotoxicity; however, clinical studies are recommended to validate our findings and also, further studies are encouraged to explore other possible molecular nephroprotective mechanisms of GB.

## Figures and Tables

**Table 1:** Experimental groups and treatment protocol in our study.

Group	Description
Group I, NC (normal control)	Rats received saline
Group II, GBC (*Ginkgo biloba* extract control)	Rats received GB by oral gavage 60 mg/kg /day for 10 days.
Group III, MTX (methotrexate)	Rats received 20 mg/kg MTX ip as a single dose on 5th day.
Group IV, GB+MTX (treated group with *Ginkgo biloba* extract and methotrexate)	Rats received GB by oral gavage 60 mg/kg/day for 10 days plus single dose 20 mg/kg MTX ip on 5th day.
